# The Effect of Silica Nanoparticles (SiNPs) on Cytotoxicity, Induction of Oxidative Stress and Apoptosis in Breast Cancer Cell Lines

**DOI:** 10.3390/ijms24032037

**Published:** 2023-01-20

**Authors:** Rafał Krętowski, Agata Jabłońska-Trypuć, Marzanna Cechowska-Pasko

**Affiliations:** 1 Department of Pharmaceutical Biochemistry, Medical University of Bialystok, Mickiewicza 2A, 15-222 Bialystok, Poland; 2 Division of Chemistry, Biology and Biotechnology, Bialystok University of Technology, Wiejska 45A, 15-351 Bialystok, Poland

**Keywords:** apoptosis, breast cancer, cytotoxicity, oxidative stress, silica nanoparticles (SiNPs)

## Abstract

Breast cancer is one of the most common cancers in women. Silica nanoparticles (SiNPs) belong to the group of often-used nanoparticles in biomedical applications. The mechanisms of the cytotoxicity, apoptosis, and oxidative stress induced by the 5–15 nm SiNPs still remain unclear. The aim of the study was to evaluate the anti-cancer effect and mechanism of action of SiNPs in breast cancer cell lines. The breast cancer MDA-MB-231 and ZR-75-1 cell lines were analyzed using MTT assay, flow cytometry, and spectrophotometric methods. In this paper, we presented findings about the cytotoxicity, apoptosis, and oxidative stress in both breast cancer cell lines. We indicated that 5–15 nm SiNPs induced dose-dependent cytotoxicity in MDA-MB-231 and ZR-75-1 cells. Moreover, we demonstrated that the process of apoptosis in the studied cell lines was associated with a decrease in the mitochondrial membrane potential (ΔΨ_m_) and an increase in the activity of caspase-9 and caspase-3. Based on the obtained results, 5–15 nm SiNPs are able to induce the mitochondrial apoptosis pathway. Analyzed nanoparticles have also been found to cause an increase in selected oxidative stress parameters in both breast cancer cell lines. The presented study provides an explanation of the possible mechanisms of 5–15 nm SiNPs action in breast cancer cells.

## 1. Introduction

The uncontrolled growth of breast cells leads to the development of breast cancer. This is one of the most common cancers in women, after lung cancer, with approximately 2 million new cases of breast cancer diagnosed in 2020. Factors that affect the formation of breast cancer are divided into two categories: modifiable and non-modifiable. Currently, most women who develop breast cancer are over the age of 50. Patient survival depends on the stage and molecular subtype of cancer. Based on mRNA gene expression levels, four basic molecular subtypes (luminal A, luminal B, HER2-enriched, and basal-like) can be distinguished among breast cancers, which allows for the implementation of new treatment strategies and patient stratifications that impact the management of breast cancer patients [1].

A growing body of scientific evidence indicates that nanotechnology can meet the growing needs of current cancer therapies. Advanced biomaterials such as nanoparticles offer a unique opportunity to maximize the effectiveness of therapy while significantly reducing its toxic side effects [2]. Silica nanoparticles (SiNPs) are among the most studied nanostructures due to their biocompatibility, large surface area for biomacromolecules, loading, relative stability, and low production costs. SiNPs are widely used as biosensors, biomarkers, and drug carriers in anti-cancer therapy [3,4]. Recently, cancer-targeted diagnostic probes, called “C-dots”, have been allowed for stage I human clinical trials [5]. C-dot probes are composed of SiNPs, and they are approved by the US Food and Drug Administration (FDA). Both in vitro and in vivo studies indicate that SiNPs induce oxidative stress, DNA destruction, and protein damage. Furthermore, the disturbances of intracellular structures caused by SiNPs may lead to apoptosis [6].

Apoptosis is a complex, multi-stage process, the inhibition of which is the basis for the development of many cancers. Actions aimed at intensifying apoptosis are the basis for the development of new therapeutic strategies [7,8,9].

The process of apoptosis is inextricably linked with changes in the level of oxidative stress, especially with an increase in the level of reactive oxygen species (ROS). ROSs cause serious damage to cellular structures and macromolecules, such as proteins (both structural and enzymatic), lipids, and nucleic acids [10]. The degree of cell damage induced by oxidative stress can be estimated by detecting selected markers of oxidative stress: the level of protein oxidation, the degree of lipid peroxidation, the GSH/GSSG ratio, and the DNA oxidation level. Oxidative damage to proteins may result in structural modifications of the polypeptide chain, which leads to changes in the structure of the native protein, and subsequently to depriving it of biological activity [10].

The reduced form of glutathione (GSH) is in close balance with the content of thiol groups in cellular proteins and protects cells from oxidative damage. One of the types of damage at the cellular level is the process of lipid peroxidation, which occurs mainly in the cytoplasmic membranes and is associated with lipid oxidation reactions. As a result of the above-mentioned chemical transformations, peroxide compounds and oxygen free radicals are formed, which activate and/or catalyze subsequent peroxidation reactions [10].

There is a scarcity of data regarding the effect of SiNPs on the mechanism of apoptosis and oxidative stress conducted in breast cancer cells as a biological model. Moreover, the mechanism of action of SiNPs on breast cancer cells is still poorly understood. Therefore, we investigated the molecular mechanism of apoptosis and proved the influence of oxidative stress on the survival of MDA-MB-231 and ZR-75-1 cell lines. The inhibitory effect of 5–15 nm SiNPs on MDA-MB-231 and ZR-75-1 cells may be associated with increased cytotoxicity, ROS generation, TBARS content, decreased GSH/GSSG ratio, and the promotion of apoptosis. In our study, two different breast cancer cell lines, MDA-MB-231 and ZR-75-1, were used. These cell lines were chosen because of their specific morphology, the activity of genes related to apoptosis, the regulation of a cell cycle, and the expression of estrogen receptors [10]. Data regarding the cytotoxicity of 5–15 nm SiNPs in breast cancer cells are very limited, and this is the first study conducted on MDA-MB-231 and ZR-75-1 cell lines.

## 2. Results

### 2.1. SiNPs Induced Cytotoxicity of MDA-MB-231 and ZR-75-1 Cells

**Figure 1:**
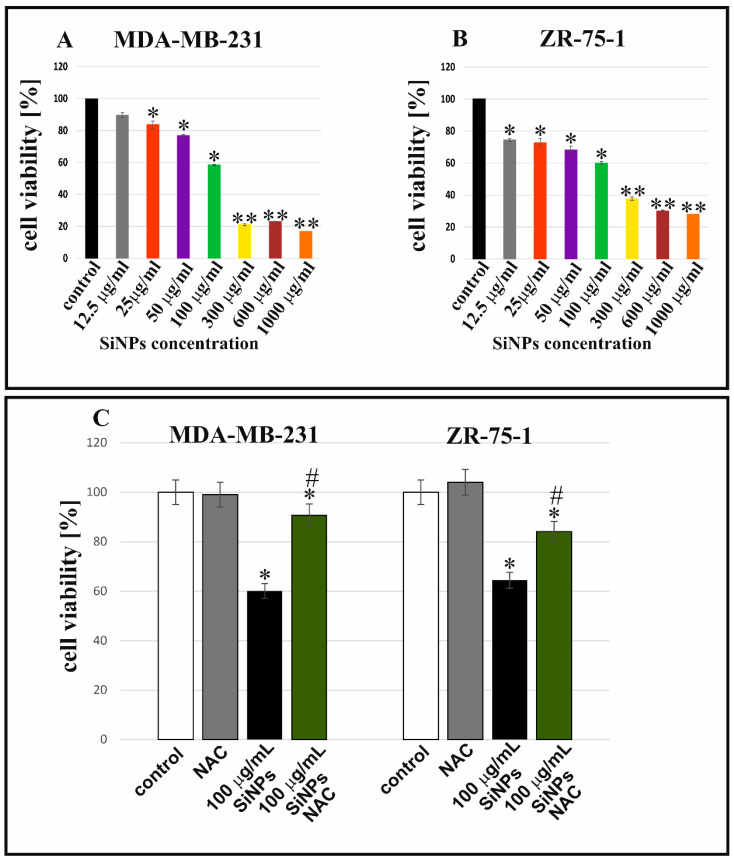
5–15 nm SiNPs induced cytotoxicity in MDA-MB-231 (**A**) and ZR-75-1 (**B**) cell lines incubated with various concentrations of 5–15 nm SiNPs: from 12.5 to 1000 µg/mL after 48 h. Bottom (**C**) shows the viability of both cell lines incubated with 100 µg/mL 5–15 nm SiNPs or 100 µg/mL 5–15 nm SiNPs + NAC (5 mmol/L), after 48 h, compared to adequate untreated controls. Mean values from three independent experiments ± SD are presented. Significant alterations are expressed relative to controls and marked with asterisks. * *p* < 0.01, *# *p* < 0.05, or ** *p* < 0.001.

Figure 1 shows the effect of 5–15 nm SiNPs on the viability of MDA-MB-231 and ZR-75-1 cell lines. The tested cells were incubated with increasing concentrations of 5–15 nm SiNPs (from 12.5 µg/mL to 1000 µg/mL). The MTT assay indicated that 5–15 nm SiNPs reduced cell viability in a dose-dependent manner. It has been shown that 5–15 nm SiNPs activated a decrease in cell viability in MDA-MB-231 (Figure 1A) and ZR-75-1 (Figure 1B) cells. The reduction in the viability of the breast cancer cells was indicated after 48 h of incubation with 5–15 nm SiNPs. A significant increase in cytotoxicity was observed when breast cancer cells were incubated with higher concentrations of 5–15 nm SiNPs. Further, we investigated the effect of antioxidant N-acetyl-L-cysteine (NAC) on the viability of MDA-MB-231 and ZR-75-1 cells. We showed that 5 mmol/L of NAC increased the viability of MDA-MB-231 and ZR-75-1 cells after incubation with 100 µg/mL 5–15 nm SiNPs + NAC in comparison to cells incubated with 100 µg/mL 5–15 nm SiNPs after 48 h (Figure 1C).

### 2.2. SiNPs Induced Apoptosis in MDA-MB-231 and ZR-75-1 Cell Lines

**Figure 2:**
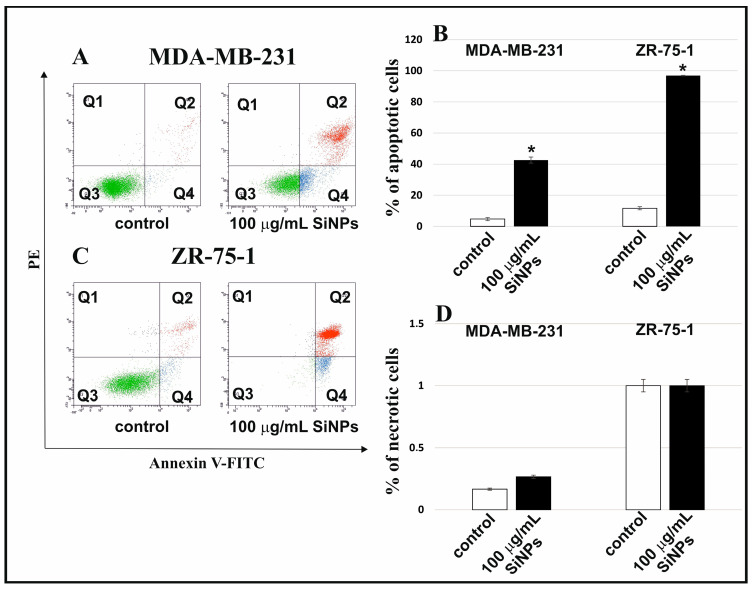
SiNPs induced apoptosis in MDA-MB-231 and ZR-75-1 cell lines. The influence of 5–15 nm SiNPs (100 µg/mL) on apoptosis (**A**–**C**) and necrosis (**A**,**C**,**D**) of both breast cancer cell lines, after 48 h. These cells were stained with Annexin V-FITC and PI. Representative dot plots for Annexin V-FITC and PI staining are shown in (**A**,**C**). Mean values from three independent experiments ± SD are presented. Significant alterations are expressed relative to adequate controls and marked with asterisks. Statistical significance was considered if * *p* < 0.001.

The apoptosis and necrosis in MDA-MB-231 and ZR-75-1 cell lines incubated with 5–15 nm SiNPs were designated using annexin V-FITC/PI double-staining by flow cytometry on FACSCanto II (BD, San Diego, CA, USA). The percentage of apoptotic and necrotic cells incubated in the medium in the presence of 100 μg/mL 5–15 nm SiNPs after 48 h is shown in Figure 2B,D. Representative dot-plots of breast cancer cells are shown in Figure 2A,C. In Figure 2B, breast cancer cell lines incubated for 48 h in the presence of 100 μg/mL 5–15 nm SiNPs were characterized by a higher percentage of apoptotic cells compared to the control culture, incubated without SiNPs. It is worth noting that ZR-75-1 cells were more sensitive to 5–15 nm SiNPs than MDA-MB-231 cells.

After 48 h of incubation with 100 μg/mL 5–15 nm SiNPs, the percent of necrotic cells was similar to the control cells.

### 2.3. The Effect of SiNPs on Mitochondria Dysfunction in MDA-MB-231 and ZR-75-1 Cells

**Figure 3:**
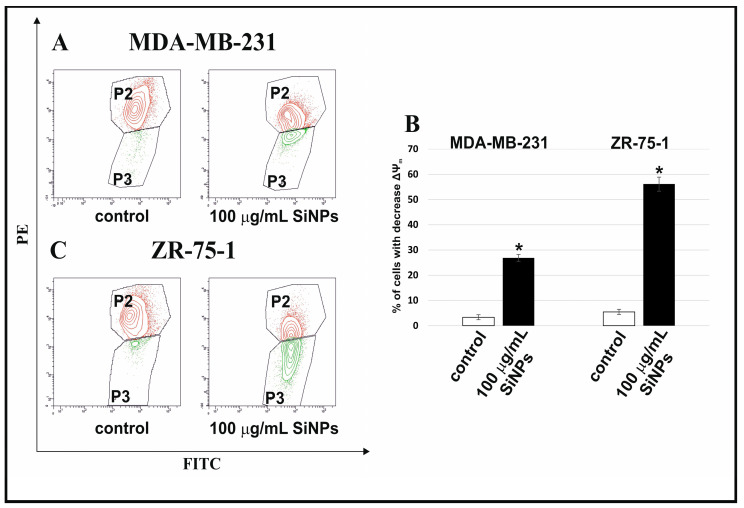
The effect of 5–15 nm SiNPs on mitochondria dysfunction in MDA-MB-231 and ZR-75-1 cells. The analyzed cells were incubated in a medium with 100 μg/mL 5–15 nm SiNPs for 48 h. The figure indicates the flow cytometry analysis of ΔΨ_m_ in MDA-MB-231 (**A**) and ZR-75-1 (**C**) cells. (**A**,**C**) show gate P2 (cells with normal mitochondrial membrane potential) and gate P3 (cells with reduced mitochondrial membrane potential). The right (**B**) shows the percentage of MDA-MB-231 and ZR-75-1 cells with decreased ΔΨm. Mean values from three independent experiments ± SD are presented. Significant alterations are expressed relative to adequate controls and marked with asterisks. Statistical significance was considered if * *p* < 0.001.

Figure 3A–C illustrates the effect of 5–15 nm SiNPs on mitochondrial membrane potential (ΔΨ_m_) in MDA-MB-231 and ZR-75-1 cells. After 48 h of incubation, a reduction in the mitochondrial membrane potential was demonstrated. The MDA-MB-231 and ZR-75-1 cells treated with 100 µg/mL 5–15 nm SiNPs showed an approximately 9-fold and 11-fold decrease in ΔΨ_m_, respectively, in comparison to the untreated cells.

### 2.4. The Effect of SiNPs on Activation of Caspase-9 and Caspase-3

**Figure 4:**
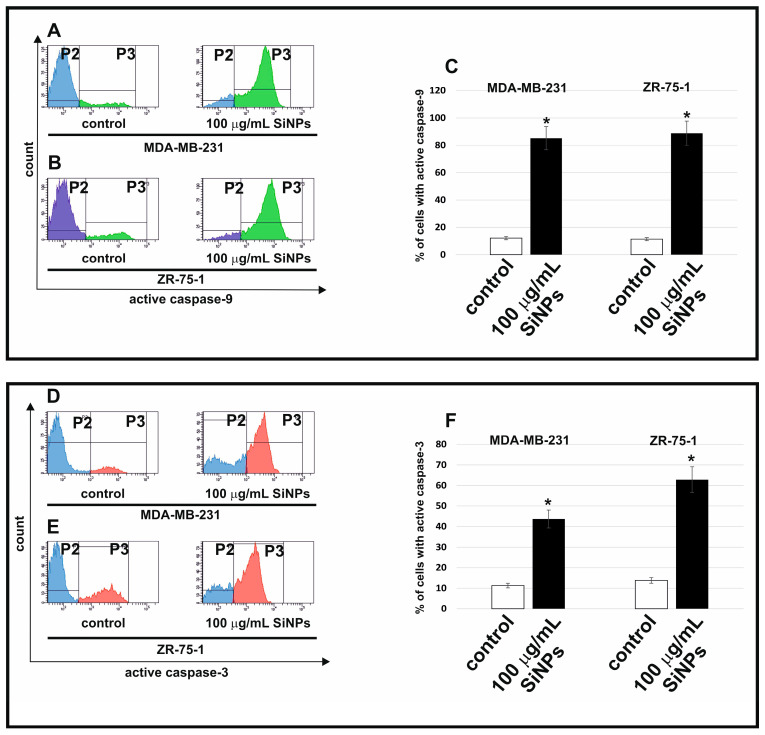
The flow cytometry analysis of active caspase-9 (**A**–**C**) and caspase-3 (**D**–**F**) in MDA-MB-231 and ZR-75-1 cell lines. The cells were incubated with 100 μg/mL 5–15 nm SiNPs for 48 h. (**A**,**B**) illustrate the histograms of MDA-MB-231 and ZR-75-1 cells-stained FAM FLICA caspase 9. (**D**,**E**) illustrate the histograms of MDA-MB-231 and ZR-75-1 cells-stained anti-Caspase-3-PE. (**C**,**F**) illustrate the percentage of MDA-MB-231 and ZR-75-1 cells with active caspase-9 and caspase-3. (**A**,**B**,**D**,**E**) show gate P2 (population of cells without active caspase-9 or active caspase-3) and gate P3 (population of cells with active caspase-9 or active caspase-3). Mean values from three independent experiments ± SD are presented. Significant alterations are expressed relative to controls and marked with asterisks. * *p* < 0.001.

Figure 4 shows the effect of 5–15 nm SiNPs on caspase-9 (A–C) and caspase-3 activation (D–F). In the current study, we examined the influence of 5–15 nm SiNPs treatment on caspase-9 and caspase-3 activation after 48 h of incubation. We showed that 5–15 nm SiNPs (100 μg/mL) increased caspase-9 and caspase-3 activity in MDA-MB-231 and ZR-75-1 cell lines compared to untreated control cells.

### 2.5. The Effect of SiNPs on Oxidative Stress

**Figure 5:**
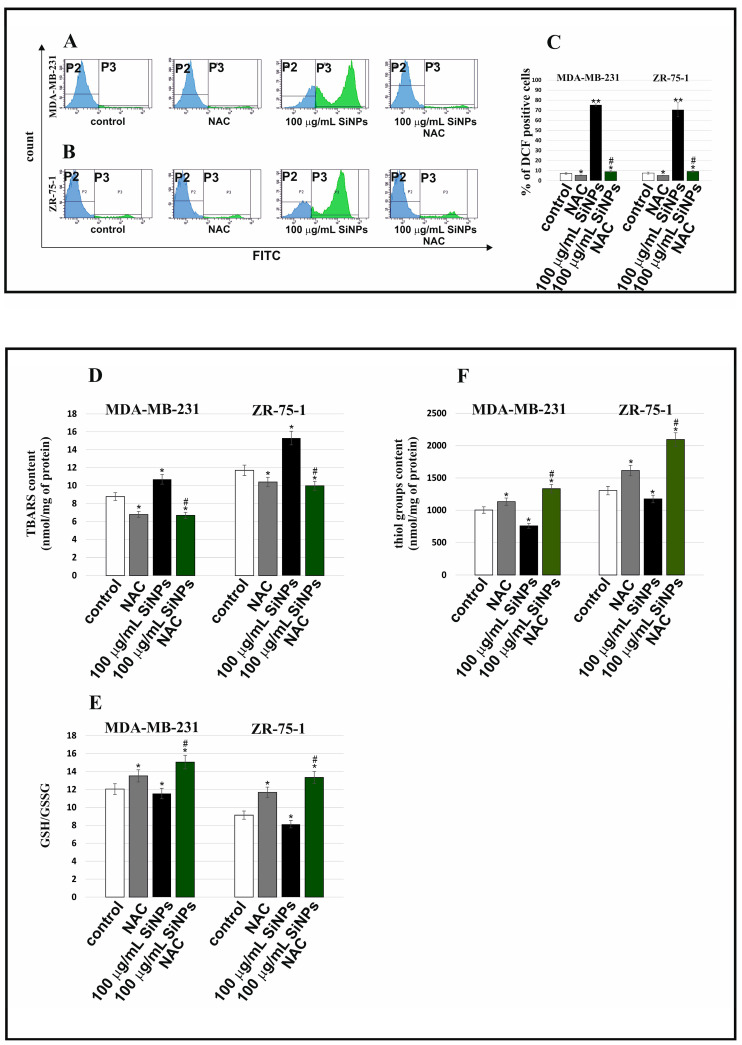
Biochemical markers of oxidative stress induced by 5–15 nm SiNPs in MDA-MB-231 and ZR-75-1 cell lines. The breast cancer cells were incubated with 100 μg/mL 5–15 nm SiNPs for 48 h. ROS production in MDA-MB-231 and ZR-75-1 cells is presented on (**A**–**C**). (**D**) shows the TBARS content. The content of thiol groups is shown on (**F)**. (**E**) shows the GSH/GSSG ratio. Data shown are the mean ± SD of triplicate experiments. Significant differences compared to the adequate control groups (* *p* < 0.01, ** *p* < 0.001 in comparison to the control of untreated cells or *# *p* < 0.05—in comparison to the control of 100 μg/mL SiNPs treated cells).

Figure 5 shows the influence of 100 µg/mL 5–15 nm SiNPs, NAC, and SiNPs with NAC on intracellular ROS production (A–C), TBARS content (D), thiol groups level (F), and GSH/GSSG ratio (E) in MDA-MB-231 and ZR-75-1 cell lines. The intracellular level of ROS production was examined by the flow cytometry method by measuring the fluorescence of DCFH-DA. The MDA-MB-231 and ZR-75-1 cells were incubated with 100 µg/mL 5–15 nm SiNPs for 48 h. SiNPs have been shown to induce an increase in the percentage of DCF-positive cells in comparison to the control cells. Preincubation of MDA-MB-231 and ZR-75-1 cells with 100 µg/mL 5–15 nm SiNPs + NAC resulted in a significant decrease in the intracellular ROS levels in comparison to the cells incubated with only 100 µg/mL 5–15 nm SiNPs.

Incubation with studied compounds caused changes in the TBARS content, which was analyzed as an index of lipid peroxidation (Figure 5D). Statistically significant changes in the tested parameter were observed in all treatments. Both NAC alone and 100 µg/mL 5–15 nm SiNPs in combination with NAC caused a decrease in the TBARS level in both studied cell lines in comparison with the control, untreated cells. However, 100 µg/mL 5–15 nm SiNPs caused an increase of about 30% in the ZR-75-1 cell line and of about 22% in MDA-MB-231 cells, as compared to adequate controls. This can be explained by the protective, anticancer properties of SiNPs manifested by an increase in the peroxidation of membrane phospholipids to such a high level that they are toxic to breast cancer cells.

The obtained results indicate that 5–15 nm SiNPs stimulate oxidative stress in breast cancer cells. The influence of 5–15 nm SiNPs and NAC on the amount of thiol groups is presented in Figure 5F. A statistically significant decrease in thiol group content, by approximately 25%, was noticed under the influence of SiNPs in the MDA-MB-231 cell line. The exposure of ZR-75-1 cells to 5–15 nm SiNPs also caused a decrease in the analyzed parameter by about 10%. However, incubation with 5–15 nm SiNPs and NAC caused a significant increase in the thiol group content in both studied cell lines. Notably, an increase was observed especially in the case of ZR-75-1 cells. The obtained results indicate that 100 μg/mL 5–15 nm SiNPs and NAC delayed the pro-oxidative effect of 5–15 nm SiNPs on breast cancer cells because we noticed an increase in thiol group content caused by 5–15 nm SiNPs and NAC in comparison to the culture treated with 100 µg/mL 5–15 nm SiNPs. The presented data may indicate that 5–15 nm SiNPs could be a compound that intensifies oxidative stress in breast cancer cells. The influence of NAC, 5–15 nm SiNPs, and the mixture of 5–15 nm SiNPs with NAC on the GSH/GSSG ratio is depicted in Figure 5E. Glutathione in its reduced form is one of the most important antioxidants whose task is to maintain the oxidative balance within the cell. A concentration of 100 µg/mL 5–15 nm SiNPs significantly decreased the GSH/GSSG ratio by about 10% in the ZR-75-1 cell line, while 100 µg/mL 5–15 nm SiNPs combined with NAC significantly increased the tested parameter by about 45%, as compared to the control. In MDA-MB-231 cell line treatment with 100 µg/mL 5–15 nm SiNPs caused a reduced ratio of GSH/GSSG by about 4% as compared to the control. The treatment with the 5–15 nm SiNPs and NAC mixture for 48 h increased the level of GSH by about 11% compared to cells treated with 5–15 nm SiNPs. Based on the obtained results, we conclude that 5–15 nm SiNPs had a rather inhibitory effect, and 5–15 nm SiNPs and NAC had a stimulating effect on the GSH/GSSG ratio in both studied cell lines.

## 3. Discussion

The mechanisms of SiNPs cytotoxicity are not fully understood. The physicochemical properties of SiNPs are the binder in research on their cytotoxicity [6]. The size characteristics of the tested 5–15 nm SiNPs were investigated in our previous study [6]. Due to their physicochemical properties and biocompatibility, 5–15 nm SiNPs are a potential candidate for biomedical applications. In our previous studies, we have shown that 5–15 nm SiNPs exhibit low toxicity to normal human skin fibroblasts [6]. In contrast, 5–15 nm SiNPs induced intracellular ROS generation, apoptosis, and autophagy in glioblastoma cells [6].

In our current research, we used the MTT assay in order to test the cytotoxic effect of 5–15 nm SiNPs on MDA-MB-231 and ZR-75-1 breast cancer cells. The results obtained in the MTT assay showed that 5–15 nm SiNPs induced a decrease in the viability of MDA-MB-231 and ZR-75-1 cells in a dose-dependent manner. Ahamed et al. showed that SiNPs induce dose-dependent cytotoxicity in both A431 and A549 cells [11], and Cui et al. indicated that silica nanoparticles can induce cytotoxicity in H9c2 cell lines [12], while Wittmaack suggested that silica nanoparticles may interfere with the cell membrane [13]. According to the literature data, SiNPs are able to induce cytotoxicity in a variety of human cell lines in a size- and dose-dependent manner [6]. Ahamed et al. indicated that SiNPs cause deprivation of the structural integrity of plasma membranes, oxidative stress, and apoptosis in human skin epithelial A431 and human lung epithelial A549 cell lines [11]. Tokgun et al. showed that the viability of A549 cell lines was reduced in a dose-dependent manner by 6 nm, 15 nm, 30 nm, and 55 nm amorphous SiNPs [14]. Another study reported that HEK293 cells incubated with 20 nm or 50 nm amorphous SiNPs exhibited a decrease in cell viability [12]. In the study of Lu et al., HepG2 cells were exposed to SiNPs, which induced cytotoxicity in a time- and dose-dependent manner [15]. Additionally, SiNPs in HepG2 cells induced oxidative stress and a mitochondrial-dependent pathway of apoptosis [15].

Although the mechanism of 5–15 nm SiNPs cytotoxicity remains unclear, it may largely depend on their physicochemical properties, such as size, shape, structure, and elemental composition [6]. In general, nanoparticles, as compared to other particles, have different physicochemical properties, such as higher surface chemical reactivity, enhanced adsorption ability, and larger specific surface area, and they may release metal ions into the cell culture environment. However, their cellular effect depends mainly on their surface reactivity and metal ion release capability, not on their “nano” size and surface area [16].

Oxidative stress and ROS generation can be induced via SiNPs [6]. According to the literature data, there are two mechanisms of inducing oxidative stress by nanoparticles: direct and indirect. In the first, ROS are generated on the surface of the nanoparticles mainly by photocatalysis and the chemical reactions of metal ions released from the nanoparticles. In turn, in the indirect mechanism, the presence of ROS is caused by mitochondrial dysfunction, which is the result of exposure to nanoparticles [16]. For this reason, we decided to study the effect of 5–15 nm SiNPs on intracellular ROS production, TBARS and thiol group content, and the GSH/GSSG ratio in breast cancer MDA-MB-231 and ZR-75-1 cells. In our study, we showed that 5–15 nm SiNPs induced ROS generation, increased TBARS content, decreased thiol group content, and decreased the GSH/GSSG ratio in both breast cancer cell lines. Additionally, we observed that pretreatment with NAC reduces oxidative stress (decreased ROS generation and TBARS content, increased GSH/GSSG ratio, and increased thiol groups content) and, interestingly, reduces cytotoxicity when breast cancer cells are treated with 5–15 nm SiNPs. These studies indicate that pretreatment of MDA-MB-231 and ZR-75-1 cells with NAC reduces the cytotoxic effect induced by 5–15 nm SiNPs, which is accompanied by the generation of ROS. NAC is the thiol group containing the ROS scavenger [17], and it is also the precursor of intracellular glutathione and cysteine, which has a free radical-scavenging ability by increasing intracellular glutathione concentrations [18]. NAC is an FDA-approved drug for the treatment of paracetamol overdose, chronic obstructive lung disease (COPD), infection, inflammation, and exposure to toxins. NAC has an anticancer effect on several types of cancer cells, e.g., breast cancer, lung cancer, colorectal cancer, bladder cancer, prostate cancer, and Kaposi’s sarcoma [18,19].

Oxidative stress is defined as an imbalance between the formation of free radicals and the abilities of individual defense mechanisms. The production of free radicals is a constant element of the cell’s oxygen metabolism. It is well known that oxidative stress causes many pathological conditions and diseases. Oxidative stress causes damage to proteins and lipids, as well as mutations and epigenetic disorders, thus leading to cancer or neurodegenerative diseases. ROS are atoms or molecules that have one unpaired electron on the valence shell. It is a broader concept than just free oxygen radicals as it also includes singlet oxygen and hydrogen peroxide. Under physiological conditions, the main source of ROS is the mitochondrial electron transport chain, in which molecular oxygen, O_2,_ is reduced to water. These reactions produce by-products, which include the superoxide anion (O^−•^), hydrogen peroxide (H_2_O_2_), hydroxyl radical (OH^•^), hydroperoxide radical (HO_2_^•^), and singlet oxygen (^1^O_2_). Depending on the concentration, ROS have the opposite effect in the cell. A moderate increase in their concentration may promote cell proliferation and survival, while exceeding a certain threshold level of ROS may lead to disturbances in the functioning of antioxidant systems, resulting in cell death [10]. Many studies have shown that SiNPs induce ROS production in different cell lines [20,21]. In vitro and in vivo studies have shown that SiNPs induce a cytotoxic effect, and overproduction of ROS mediates oxidative stress and contributes to SiNPs-induced cell damage [22]. Cui et al. demonstrated that SiNPs induced ROS generation and decreased superoxide dismutase (SOD), glutathione (GSH), and GSH-peroxidase (GPx) activity. Apart from that, incubation with NAC decreases the release of LDH and ROS while increasing the activity of SOD, GSH, and GPx [12]. Guo et al. showed that oxidative stress induced via ROS generation leads to redox imbalance and lipid peroxidation [22]. Petrache Voicu et al. demonstrated strong oxidative stress, protein damage, and a reduction in GSH level, which was observed in MRC-5 cell lines during incubation with SiNPs [23]. We suggest that a significant increase in ROS synthesis, production of TBARS, and decrease in GSH/GSSG ratio can attest to strong oxidative stress, which induces cytotoxicity and apoptosis in test cells incubated in the presence of 5–15 nm SiNPs.

Many studies showed that apoptosis and necrosis are the main types of cell death [6,7,10]. Programmed cell death is one of the most important pathways for maintaining homeostasis in conditions of cell division or death. Dysregulation of apoptosis leads to the induction of most cancers. The search for new therapeutic strategies in the fight against cancer involves the implementation of nanotechnology for in vitro research [15]. To investigate the consequences of the cytotoxic effect of 5–15 nm SiNPs, apoptosis was measured using the flow cytometry method with Annexin V-FITC/PI staining. Our research indicates that 5–15 nm SiNPs induce apoptosis in MDA-MB-231 and ZR-75-1 cell lines. Lu et al. showed that 7–20 nm SiNPs induced oxidative stress and apoptosis in HepG2 cell lines. Moreover, the SiNPs showed low cytotoxicity in the L-02 control cells [15]. This is in line with our previous studies, where we demonstrated low cytotoxicity of 5–15 nm SiNPs in human skin fibroblast cells [6].

Several studies indicated that SiNPs can be transported into the cell by diffusion, while larger SiNPs either settle on the cell surface or are absorbed by endocytosis. Smaller SiNPs can penetrate the cell interior and induce oxidative damage to the mitochondrial membrane, while the largest SiNPs tend to induce membrane injury by mechanical friction or oxidative damage [23].

Numerous pro-apoptotic factors are released into the cytosol through the permeabilization of the outer mitochondrial membrane. These factors promote caspase activation. Next, cytochrome c binds to apoptotic protease activating factor-1 (Apaf-1) and leads to the assembly of the apoptosome complex [11].

In the activation of an intrinsic pathway of apoptosis in human/mammalian cells, an important role is played by mitochondria [6]. We obtained results indicating a significant loss of ΔΨ_m_, which was observed especially in ZR-75-1 cells. Previously, we presented that 5–15 nm SiNPs induced the loss of ΔΨ_m_ and changes in the mitochondrial ultrastructure, and we also proved that 5–15 nm SiNPs activated apoptosis in LBC3 glioma cells via the mitochondrial pathway [6]. According to Ahamed et al., silica nanoparticles caused the opening of mitochondrial permeability transition pores (MPTP) followed by the loss of ΔΨ_m_ inducing ROS production and apoptosis [11]. Therefore, we decided to estimate the influence of 5–15 nm SiNPs on caspase-9 and caspase-3 activity in MDA-MB-231 and ZR-75-1 cell lines.

In summary, in the conducted research, we find a similar mechanism of action of SiNPs on MDA-MB-231 and ZR-75-1 cells in comparison with Ahamed et al.’s study on human skin epithelial A431 and human lung epithelial A549 cell lines. They investigated the effect of SiNPs in the concentration range of 25 to 200 μg/mL [11]. In our research on breast cancer cells, we extended the concentration range to 1000 μg/mL SiNPs. Further, Yuji investigated the effects of SiNPs on hippocampal cells. Contrary to our research, they used a much larger size range of SiNPs, as far as 1500 nm. As in our research, scientists have shown that SiNPs induce oxidative stress and apoptosis. Moreover, they found that the cytotoxicity was dependent on the SiNPs’ size, concentration, and surface charge [24]. Abo El-Maali showed that silicon nanoparticles in combination with flavone compounds exhibit cytotoxic activity on MDA-MB-231 and MCF-7 cells but not on a non-tumorigenic epithelial cell line isolated from the mammary gland MCF-10 cell line. Additionally, using annexin V, propidium iodide double-staining followed by flow cytometry analysis, they found that the combination of flavones with NP significantly induced apoptosis in MCF-7 and MDA-MB-231 cancer cell lines [25].

**Figure 6:**
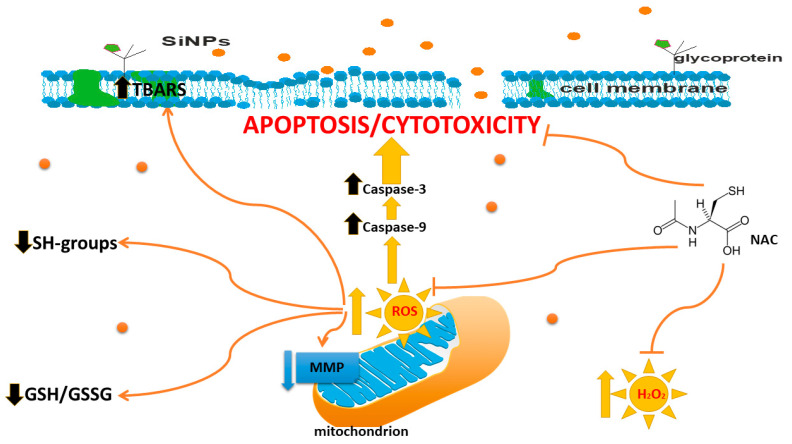
The effect of 5–15 nm SiNPs on cytotoxicity, apoptosis, and oxidative stress in MDA-MB-231 and ZR-75-1 cell lines.

Our research showed that 5–15 nm SiNPs activated caspase-9 and caspase-3. Subsequently, the apoptosome binds to caspase-9, and it goes on to activate caspase-3, which appears to be an event common in SiNPs-induced apoptosis. Ahamed et al. showed that silica nanoparticles increased caspase-3 and caspase-9 activity in A431 and A549 cells [11]. Consistent with this observation, our research, presented above, suggest that 5–15 nm SiNPs induced cytotoxicity, ROS generation, an increase in TBARS content, a decrease in GSH/GSSG ratio, and an increase in apoptosis of both MDA-MB-231 and ZR-75-1 breast cancer cells (Figure 6).

## 4. Materials and Methods

### 4.1. Reagents

Media for cells cultivation: L-15 Medium (1×) + GlutaMAX^TM^-I, trypsin-EDTA, RPMI Medium 1640 (1×) + GlutaMAX^TM^-I, DPBS, penicillin, streptomycin, and fetal bovine serum Gold (FBS Gold) were provided by Gibco (San Diego, CA, USA).

Agents for apoptosis detection: JC-1 MitoScreen Kit, PE Active Caspase-3 Apoptosis Kit, and FITC Annexin V apoptosis detection Kit I were obtained from BD Pharmingen^TM^ (San Diego, CA, USA); FAM-FLICA Caspase-9 Assay was provided by ImmunoChemistry Technologies (Davis, CA, USA). Chemical treatment of cells: silica nanoparticles 5–15 nm (SiNPs) were obtained from Sigma-Aldrich (St. Louis, MO, USA).

Chemical compounds for oxidative stress analysis: SDS, TCA, TBA, Folin-Ciocalteu reagent, N-acetylcysteine, and 2’,7’-dichlorodihydrofluorescein diacetate (H_2_DCFDA) were provided by Sigma-Aldrich (St. Louis, MO, USA) and GSH/GSSG-Glo^TM^ Assay kit by Promega (Madison, WI, USA).

### 4.2. Cell Culture

Both human breast cancer cell lines, MDA-MB-231 and ZR-75-1, were obtained from American Type Culture Collection (ATCC). Cells were cultured as described in our previous paper [10].

### 4.3. Chemical Treatment of Cells

To avoid the problem with the aggregation of 5–15 nm SiNPs, 5–15 nm SiNPs were dispersed in deionized water (1 mg/mL) by a sonicator, Sonopuls (Bandelin, Berlin, Germany), on ice for 45 min (160 W, 20 kHz). During experiments, cell culture media were replaced with a new one containing 5–15 nm SiNPs suspensions, at concentrations ranging from 12.5 μg/mL to 1000 μg/mL. Negative control constituted non-treated cells. For the analysis of cell viability, the 5–15 nm SiNPs concentration range was from 12.5 to 1000 µg/mL. In a separate MTT and oxidative stress experiment, cells were pre-treated (for 1 h) with NAC (5 mmol/L). Subsequently, cells were exposed to 5–15 nm SiNPs for 48 h. Apoptosis and necrosis detection and mitochondrial membrane potential (∆Ψm) analysis were conducted under the influence of 5–15 nm SiNPs at 100 μg/mL concentration. For the analysis of caspase-9 enzymatic activity, cells were treated with 100 µg/mL 5–15 nm SiNPs for 48 h. In the caspase-3 assay, cells were treated with 100 µg/mL of 5–15 nm SiNPs for 48 h.

### 4.4. Cell Viability

Cell viability was measured based on the methods of Carmichael et al. using 3-(4,5-dimethylthiazol-2-yl)-2,5-diphenyltetrazolium bromide (MTT) as described in our previous studies [17].

### 4.5. Detection of Apoptosis and Necrosis

Detection of apoptosis and necrosis was conducted with the use of FACSCanto II flow cytometry (BD, San Diego, CA, USA) as described in our previous studies [17].

### 4.6. Mitochondrial Membrane Potential (∆Ψm) Analysis

Disruption of the mitochondrial membrane potential was analyzed with the use of the MitoScreen kit (BD, San Diego, CA, USA), according to the manufacturer’s guidelines as it was described previously [17].

### 4.7. Caspase-9 Enzymatic Activity Assay

Caspase-9 activity was measured using the FAM-FLICA Caspase 9 Kit (ImmunoChemistry Technologies) according to the manufacturer’s instructions as described in our previous studies [17].

### 4.8. Caspase-3 Assay

Caspase-3 was measured using the PE Active Caspase-3 Apoptosis Kit according to the manufacturer’s instructions as described in our previous studies [17].

### 4.9. Intracellular ROS Detection by Flow Cytometry

The level of intracellular ROS was determined using a dichlorodihydrofluorescein diacetate (DCFH-DA) assay, (Sigma, St. Louis, MO, USA) as described in our previous studies [7].

### 4.10. Determination of SH Groups

SH groups were measured using the method of Rice-Evans [26], as described previously by Jabłońska-Trypuć et al. [27]. The MDA-MB-231 and ZR-75-1 cells (2.5 × 10^5^ cells/mL) were incubated in 2 mL of medium with or without the test 5–15 nm SiNPs (100 μg/mL) in 6-well plates.

### 4.11. Determination of TBA Reactive Species (TBARS) Levels

The level of membrane lipid-peroxidation products, or TBARS, was measured using the method of Rice-Evans [26], as described previously by Jabłońska-Trypuć et al. [27]. The MDA-MB-231 and ZR-75-1 cells (2.5 × 10^5^ cells/mL) were incubated in 2 mL of medium with or without the test 5–15 nm SiNPs (100 μg/mL) in 6-well plates.

### 4.12. Determination of GSH/GSSG

The total glutathione content and GSH/GSSG ratio were studied with the use of a GSH/GSSG-Glo™ kit (Promega Madison, WI) following the manufacturer’s instructions. Cells of both cell lines were cultured in white 96-well plates and exposed to 5–15 nm SiNPs (100 μg/mL).

Prior to the assay, growth media were removed, and cells were washed with PBS. The assay is based on a luminescence measurement and detects and quantifies total glutathione (GSH +GSSG), GSSG, and GSH/GSSG ratios in cultured cells. Stable luminescent signals are correlated with either the GSH or GSSG concentration of a sample. In this method, the GSH-dependent conversion of a GSH probe, Luciferin-NT, to luciferin by a glutathione S-transferase enzyme is coupled to a firefly luciferase reaction. The light from luciferase depends on the amount of luciferin formed, which in turn depends on the amount of GSH present. Thus, the luminescent signal is proportional to the amount of GSH. GSH/GSSG ratios are calculated directly from the luminescence measurements.

### 4.13. Determination of Total Protein Concentration

The MDA-MB-231 and ZR-75-1 cells (2.5 × 10^5^ cells/mL) were incubated in 2 mL of medium with or without the test compound in 6-well plates. The cells were then homogenized at 4 °C, and the total protein concentration in 0.1 M NaOH extract was calculated. Protein concentration was determined spectrophotometrically according to the method of Lowry et al [28]. The absorbance of the extracts was measured spectrophotometrically at 750 nm [28].

### 4.14. Statistical Analysis

Mean values from three independent experiments ± standard deviations (SD) were calculated. The data were statistically analyzed using one way-ANOVA followed by Tukey’s post hoc *t*-test analysis. The significant differences of means were determined at the level of * *p* < 0.05 or ** *p* < 0.001.

## 5. Conclusions

In summary, the present study indicated that 5–15 nm SiNPs induced cytotoxicity and apoptosis in both studied cell lines. The level of apoptosis in MDA-MB-231 and ZR-75-1 cells was correlated with an observed decrease in ΔΨ_m_ and an increase in the activity of caspase-9 and caspase-3. Obtained results indicate that 5–15 nm SiNPs are able to induce the mitochondrial pathway of apoptosis. However, 5–15 nm SiNPs have also been found to cause an increase in the oxidative stress level in MDA-MB-231 and ZR-75-1 cells (Figure 6). The presented results demonstrate that pretreatment of breast cancer cells with NAC ameliorates the oxidative stress stimulated by 5–15 nm SiNPs.

Taken together, our results may contribute to a better understanding of the coexistence of two phenomena, apoptosis and oxidative stress, and their possible role in breast cancer treatment. The novelty of the presented study consists mainly in analyzing the possible mechanisms of 5–15 nm SiNPs cytotoxic action and their role in the induction of apoptosis. Therefore, we suggest that 5–15 nm SiNPs may be a useful tool in the treatment of breast cancer.

## Data Availability

Not applicable.
